# A Combined Supplement of Probiotic Strains AP-32, bv-77, and CP-9 Increased *Akkermansia mucinphila* and Reduced Non-Esterified Fatty Acids and Energy Metabolism in HFD-Induced Obese Rats

**DOI:** 10.3390/nu14030527

**Published:** 2022-01-26

**Authors:** Chorng-An Liao, Cheng-Hsieh Huang, Hsieh-Hsun Ho, Jui-Fen Chen, Yi-Wei Kuo, Jia-Hung Lin, Shin-Yu Tsai, Hui-Yun Tsai, Yao-Tsung Yeh

**Affiliations:** 1 Aging and Diseases Prevention Research Center, Fooyin University, Kaohsiung 83102, Taiwan; andyliao1993@gmail.com (C.-A.L.); prevailingkimo@gmail.com (C.-H.H.); 2 Biomed Analysis Center, Fooyin Hospital, Pingtung 92847, Taiwan; 3 Ph.D. Program in Environmental and Occupational Medicine, Kaohsiung Medical University, Kaohsiung 80708, Taiwan; 4 Department of Research and Design, glac Biotech Co., Ltd., Tainan 74442, Taiwan; sam.ho@bioflag.com.tw (H.-H.H.); juifen.chen@bioflag.com.tw (J.-F.C.); vic.kuo@bioflag.com.tw (Y.-W.K.); jiahung.lin@bioflag.com.tw (J.-H.L.); Shin-Yu.Tsai@bioflag.com.tw (S.-Y.T.); 5 Department of Nutrition and Health Science, Fooyin University, Kaohsiung 83102, Taiwan; 6 Department of Medical Laboratory Sciences and Biotechnology, Fooyin University, Kaohsiung 83102, Taiwan

**Keywords:** obesity, dysbiosis, fat accumulation, probiotics, *Akkermansia muciniphila*

## Abstract

Obesity is referred to as a condition in which excess body fat has accumulated to an extent that it causes negative impacts on health. The formation of body fat is regulated by complicated networks in relation to energy metabolism, and gut microbiota have been regarded as a key player. Studies have shown that supplements of probiotics provide benefits to health, including an improvement in metabolic syndrome and the control of body weight. In the present study, three probiotic strains, AP-32, bv-77, and CP-9, stood out from nine candidates using a lipid consumption assay, and were subsequently introduced to further animal tests. A rodent model of obesity was induced by a high-fat diet (HFD) in Sprague-Dawley (SD) rats, and three probiotic strains were administered either separately or in a mixture. A low dose (5 × 10^9^ CFU/kg/day) and a high dose (2.5 × 10^10^ CFU/kg/day) of probiotics were orally provided to obese rats. The bioeffects of the probiotic supplements were evaluated based on five aspects: (1) the body weight and growth rate; (2) ketone bodies, non-esterified fatty acids (NEFAs), and feed efficiency; (3) blood biochemistry; (4) fat content; and (5) gut microbiota composition. Our results demonstrated that the supplement of AP-32, CP-9, and bv-77 alleviated the increasing rate of body weight and prevented the elevation of NEFAs and ketone bodies in obese rats. Although the effect on fat content showed a minor improvement, the supplement of probiotics displayed significant improvements in HFD-induced poor blood biochemical characteristics, such as alanine aminotransferase (ALT), aspartate Transaminase (AST), and uric acid, within 4 weeks. Furthermore, the combined supplement of three strains significantly increased *Akkermansia mucinphila* as compared with three individual strains, while its enrichment was negatively correlated with NEFAs and energy metabolism. In general, a mixture of three probiotic strains delivered a better outcome than a single strain, and the high dose of supplements provided a more profound benefit than the low dose. In conclusion, three probiotic strains, AP-32, bv-77, and CP-9, can alleviate body fat formation in obese rats. Furthermore, a combined supplement of these three probiotic strains may have potential in treating or controlling metabolic disorders.

## 1. Introduction

Body mass index (BMI) has been used by the World Health Organization (WHO) as the standard for recording obesity statistics since the early 1980s, and obesity has been listed as a chronic disorder by the WHO and U.S. Food and Drug Administration (FDA) since 1996. Studies have found that the association between mortality risk and BMI value is different among the human race, and the risk of negative health effects begins to increase between 22 and 25 kg/m^2^ in Asians [1]. Obesity also increases the risk of many physical conditions, such as type two diabetes, high blood pressure, high blood cholesterol, and high triglyceride levels [2]. Obesity causes not only physical problems but also social problems. Some research shows that obese people are less likely to be hired for a job and are less likely to be promoted [3]. A combination of excessive food intake and a lack of exercise is thought to explain most cases of obesity; however, the modern lifestyle has ironically created more possible contributors to the recent increase in obesity, such as insufficient sleep and environmental pollutants [4].

Scientists have been working to combat obesity for more than 20 years; however, the obese population has become larger rather than smaller [5]. Obesity can be classified into two types: simple obesity and secondary obesity; the only rare type being secondary obesity, which is mainly disease-related or medical-related [6]. Most of the cases are simple obesity, which can be caused by increased amounts of fat cells or by increased volumes of fat cells [7]. In order to maintain a healthy BMI, many food supplements have been designed to disrupt body fat formation by either regulating gut functions or activating hormone-sensitive lipase. Hormone-sensitive lipase exists in fat tissues and promotes the degradation of triglyceride (TG) when activated [8]. Although body fat formation is regulated by complicated networks, the digestion of fats in the intestine is crucial. The absorption of lipids decreases when the digestion of fats in the intestine is inhibited [9].

Gut microbiota symbiotically reside in human digestive tracts, and probiotics, as defined by the WHO in 2001, are bacteria strains which have beneficial effects on human health. Populations of probiotics are dynamically changed by the daily life of an individual and severely affected by the mental and physical conditions of the host [10]. An unhealthy lifestyle can tilt the balance of the microbiome, and an imbalance in the composition of the microbiota may cause sub-health conditions, such as obesity, at the early stage [11]. Increasingly more evidence has proven the role of gut microbiota in energy metabolism [12,13], and the *Lactobacillus rhamnosus GG* strain has been reported to consume fatty acids during cultivation in vitro [14]. An unhealthy diet containing a high amount of fat or sucrose reduces gut microbiota diversity and can lead to an alteration in the bacterial composition related to obesity [15]. Non-esterified fatty acids (NEFAs), also called free fatty acids (FFAs), elevate in the blood when adipose tissue mass increases and initiate many adverse metabolic effects [16]. Paralleling the obesity epidemic, higher circulating ketone bodies are associated with an elevated fatty liver index (FLI), which is highly correlated to non-alcoholic fatty liver disease (NAFLD) [17]. Hepatocytes uptake about 60% of fatty acids as an energy source in NFALD patients [18]. Energy metabolism in the liver is delicately controlled. The dysregulation of liver signaling and metabolism may promote NAFLD and type two diabetes [19]. It is notable that NEFAs are produced and released from adipocytes, beta-oxidized in the hepatocytes, and then generate ketone bodies [17]. In high-fat diet (HFD)-induced obese mice models, it was found that gut dysbiosis reduced *Akkermansia mucinphila* in obese mice [20] and children with NAFLD [21]. *Akkermansia mucinphila*, however, was difficult to maintain in vitro and survive as a probiotic [22]. Promoting *Akkermansia mucinphila* in the gut will be an important goal to achieve.

In this study, *Lactobacillus salivarius* AP-32, *Lactobacillus rhamnosus* bv-77, and *Bifidobacterium animalis* CP-9 were the three best out of nine probiotic strains screened by a lipid consumption assay in vitro. The symptom of obesity was induced by high-fat diets (HFDs) in SD rats before probiotic intervention. The obese rats were continuously fed HFDs and treated with two different dosages, low and high, of probiotic strains. Three probiotic strains: AP-32, bv-77, and CP-9, were administered either separately or all together in a mixture. Body weight was recorded every 2 weeks, and blood biochemistry as well as body fat formation were analyzed after probiotic intervention. To visualize the accumulation of body fat, animals were euthanized and tissue fats were measured at the end of the experiment. Our results demonstrated that the supplement of AP-32, CP-9, and bv-77 alleviated the increasing rate of body weight and prevented the elevation of non-esterified fatty acids (NEFAs) as well as ketone bodies in obese rats within 4 weeks. A combined supplement of three strains significantly increased *Akkermansia mucinphila*, which was negatively correlated with non-esterified fatty acids and energy metabolism. Our results provide an indirect strategy that could effectively increase the so-called next-generation probiotic, *Akkermansia mucinphila*, and may have potential in treating and controlling obesity-related metabolic disorders.

## 2. Materials and Methods

### 2.1. Rats

Male SD rats were purchased from BioLASCO Taiwan Co., Ltd. (Taipei, Taiwan) and housed with 2 rats per cage at the Laboratory Animal Center, National Chiao Tung University. The animals were fed with sterilized water and foods. The breeding environment was well-monitored and well-controlled (12 h light/dark cycle, 22 ± 2 °C, and 62 ± 5% humidity). Animal experiments and protocols were in compliance with the Laboratory Animal Care and Use Guidelines published by the Government of Taiwan. The protocols were approved by the National Chiao Tung University Animal Ethics Committee (approval no. NCTU-IACUC-107031).

### 2.2. HFD-Induced Obese Animal Model

A Harlan^®^ Diet-Induced Obesity high-fat diet (HFD) was used in all experimental animals except the blank control (C) group (Research Diets Inc, D12451). The fat content of the fodder formula was 45% of its total calories. Fasting blood glucose levels were monitored every 2 weeks in reference to untreated rats (C). Body weight was measured every 2 weeks. The percentage of weight gain was normalized to the body weight at week 0.

### 2.3. Probiotic Treatments

Three probiotic strains, AP-32, bv-77, and CP-9, stood out from nine candidates using a lipid consumption assay (Figure S1), and were subsequently introduced to further animal tests. It is noted that *Lactobacillus salivarius* AP-32 was isolated from the human gut, and *Lactobacillus rhamnosus* bv-77 as well as *Bifidobacterium animalis* subsp. *lactis* CP-9 were isolated from breast milk. These probiotic strains were provided by Glac Biotech Co., Ltd. (Tainan, Taiwan). Lyophilized probiotic powder was diluted in PBS (phosphate-buffered saline) and fed to animals by oral gavage. Rats were fed a high-fat diet from weeks 0 to 4, and obese rats were given 1000 μL oral gavage daily from week 5 to week 8. The oral gavage contained a low or high dose (low dose, L = 5 × 10^9^ CFU/kg/day, or high dose, H = 2.5 × 10^10^ CFU/kg/day) of a single probiotic strain or a mixture (MIX) of three strains in experimental groups. The blank control (C) group was fed a standard diet and given oral gavage containing PBS without probiotics. The experimental control (CH) group was fed an HFD and given oral gavage containing PBS without probiotics. Eight probiotic groups were set up as AP-32(L), bv-77 (L), CP-9 (L), MIX (L), AP-32 (H), bv-77 (H), CP-9 (H), and MIX (H). The animals were euthanized at the end of the treatment. Serum and fat tissue samples were collected for biochemistry and body fat accumulation analyses. Six rats were examined in each group.

### 2.4. Glycemic Level Investigation

Fasting blood glucose level was monitored every 2 weeks; the blood sample was collected from the tail of the rat 12 h after the removal of the food tray from the cage. The glycemic level was measured by using an EasyTouch GCU Blood Glucose/Cholesterol/ Uric Acid Multi-Function Monitoring System No. ET-301 (Bioptik Technology, Inc., Miaoli, Taiwan) and blood glucose test strip No. 3372.

### 2.5. Serum Biochemistry

Blood samples were immediately centrifuged after collection and stored at −80 °C until evaluation. Serum aspartate aminotransferase (AST), alanine transaminase (ALT), uric acid (UA), ketone bodies (KB), creatinine (CREA), sodium (Na^+^), potassium (K^+^), total cholesterol (CHOL), low-density lipoprotein (LDL), high-density lipoprotein (HDL), triglyceride (TG), and non-esterified fatty acid (NEFA) were evaluated by using available test strips with an Automated Clinical Chemistry Analyzer (FUJIFILM, DRI-CHEM 4000i, Tokyo, Japan).

### 2.6. 16S rRNA Sequencing of Fecal Sample

Fecal samples were collected after probiotic treatments and all fecal specimens were extracted using a Qiagen DNA kit (Hilden, Germany) according to the manufacturer’s instructions. DNA samples with optical density (OD) 260/280 in the range of 1.8~2.0. Using metagenomic DNA as a template, 16S rDNA PCR was amplified with the bacterial-specific primers 314F (5′-TCG TCG GCA GCG TCA GAT GTG TAT AAG AGA CAG CCT ACG GGN GGC WGC AG-3′) and 805R (5′-GTC TCG TGG GCT CGG AGA TGT GTA TAA GAG ACA GGA CTA CHV GGG TAT CTA ATC C-3′). The amplified DNA sizing was checked by TapeStation (Agilent Technologies, Santa Clara, CA, USA). Sequencing was carried out by the Illumina Miseq platform. Indices and Illumina sequencing adapters were attached to DNA samples using a Nextera XT Index Kit (Juno Beach, FL, USA). After library construction, samples were mixed with MiSeq Reagent Kit v3 (600-cycle, Illumina, San Diego, CA, USA) and loaded onto a Miseq cartridge, then onto the instrument. Automated cluster generation and a 2 × 300 bp paired-end sequencing run was performed. The sequences generated went through a filtering process to obtain the qualified reads. Total reads were merged after removing low-quality sequences and chimeric sequences, and clustered to operational taxonomic unit (OTU)-at 97% similarity with the Greengenes database (v13.8). All OTU sequences and diversity analysis were obtained using a CLC Microbial Genomics Module (v10.0, Qiagen, Germany), BaseSpace (Illumina, San Diego, CA, USA), and GraphPad Prism 8 (GraphPad Software, San Diego, CA, USA). A *p*-value less than 0.05 is considered statistically significant.

### 2.7. Bioinformatics Analysis

Statistical analysis was performed using a CLC Microbial Genomics Module. The alpha diversity was measured using the Shannon index, which calculates the overall diversity of each group, including the number of observed species (richness) and how evenly the observed taxonomies are seen (evenness). Beta diversity was measured using PCoA-weighted UniFrac, which determines the difference in microbial composition between groups. Hierarchical clustering of the top 25 abundant taxonomies were deduced using a heatmap to determine patterns between a group out OTU table that was generated by a CLC Microbial Genomics Module to be further analyzed with Linear discriminant analysis Effect Size (LEfSe) and Phylogenetic Investigation of Communities by Reconstruction of Unobserved States (PICRUSt) analysis. LEfSe was performed via the website of Galaxy/HutLab (http://huttenhower.sph.harvard.edu/galaxy/, 20 December 2021) to identify specific microbial markers between groups with an alpha value for the factorial Kruskal–Wallis test/pairwise Wilcoxon test of 0.05 and an LDA (Linear discriminant analysis) score cut-off of 2.0. PICRUSt prediction was performed via the Galaxy website according to the Kyoto Encyclopedia of Genes and Genomes (KEGG) functional pathways database and analyzed with Statistical Analysis of Metagenomic Profiles (STAMP, v2.1.3, https://beikolab.cs.dal.ca/software/STAMP, 20 December 2021) software. The criteria of STAMP were set up with removed unclassified reads, *p* < 0.01, and effect size 0.2. The results revealed the functional pathways with a significantly different abundance at level 3 between groups. Spearman’s correlation and principal component analysis (PCA) were utilized by the R language (v4.0.2, *R Core Team 2020*). The comparison of different groups was performed by a two-tailed t-test. A *p*-value less than 0.05 was considered statistically significant.

### 2.8. Statistical Analysis

Statistical analysis was also performed using Microsoft Excel software (Redmond, WA, USA) and GraphPad Prism software (San Diego, CA, USA). Graphs and tables represent mean ± standard deviations (SDs) obtained from two or three independent experiments and collected from 6 animals. Differences were evaluated using one-way ANOVA. Multiple comparison was performed by Duncan’s new multiple range test (MRT). Results with *p*-values of less than 0.05 were considered significant.

## 3. Results

### 3.1. The Supplement of AP-32, bv-77, and CP-9 Alleviated Weight Gain in Obese Rats

**Figure 1:**
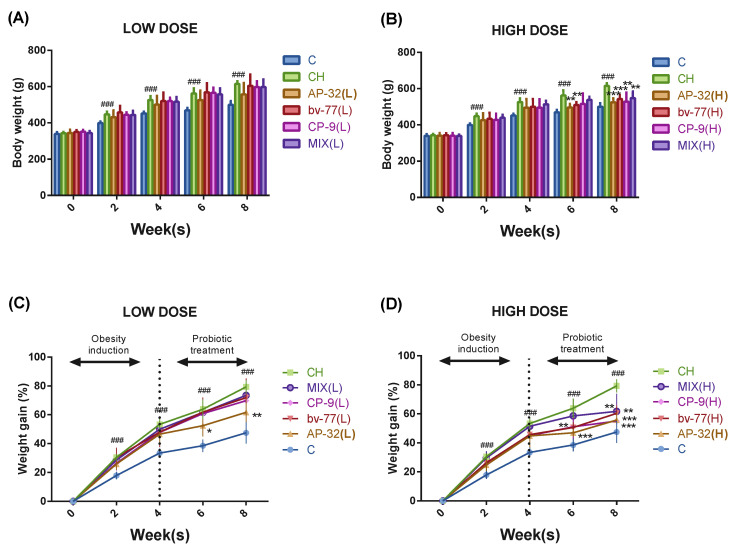
Body weights and weight gain rates in obese rats from week 0 to week 8. Sprague-Dawley (SD) rats were fed a high-fat diet to induce obese symptoms for 8 weeks. The probiotic groups were treated with a single strain (AP-32, bv-77, or CP-9) or a mixture of 3 strains (MIX) at a low (L = 5 × 10^9^ CFU/kg/day) or high (H = 2.5 × 10^10^ CFU/kg/day) dosage. The group of rats fed a normal diet was the blank control (C), and the one fed a high-fat diet was the symptom control (CH). The bar graph represents the body weights of rats treated with a (**A**) low dose and (**B**) high dose of a probiotic strain(s). The weight gain rate was normalized to the body weight at week 0, and presented as a percentage in rats treated with a (**C**) low dose and (**D**) high dose of a probiotic strain(s). The body weight was recorded every 2 weeks, and data show the mean ± S.D. of six animals in each group. The dotted line represents the beginning of the probiotic supplementation. Statistical analysis was performed by using a one-way ANOVA. Statistical difference was compared between the C and CH groups (^###^ *p* < 0.005) or between the CH and a probiotics-treated group (* *p* < 0.05, ** *p* < 0.01, and *** *p* < 0.005).

At week 0, the body weights of all the rats were similar before the induction of the high-fat diet (HFD), and the HFD treatment induced the symptom of obesity in 2 weeks. Both body weight and weight gain increased significantly (^###^ *p* < 0.001) in the symptom control group (CH) compared to the blank control group (C) from week two to week eight (Figure 1). Obesity was induced before probiotic interventions from week one to week four, and probiotic strains were provided together with the HFD from week five to week eight. At week six, the body weight was 469.40 ± 13.90 g in the C group and 562.12 ± 28.14 g in the CH group. At week eight, the body weight was 499.40 ± 20.90 g in the C group and 614.92 ± 14.71 g in the CH group. Compared to the CH group at week six, body weights were significantly (** *p* < 0.01) lower in the AP-32(H) and bv-77(H) groups, and were significantly (** *p* < 0.01, *** *p* < 0.001) lower in the AP-32(H), bv-77(H), CP-9(H), and MIX(H) groups at week eight (Figure 1A,B). At week six the weight gain rate was 38.55 ± 4.33% in the C group and 63.85 ± 6.68% in the CH group. At week eight the weight gain rate was 47.45 ± 7.42% in the C group and 79.30 ± 4.46% in the CH group. Compared to the CH group at week six, the weight gain rates were significantly (* *p* < 0.05, ** *p* < 0.01, and *** *p* < 0.001) lower in the AP-32(L), AP-32(H), and bv-77(H) groups, and were significantly (** *p* < 0.01, *** *p* < 0.001) lower in the AP-32(L), AP-32(H), bv-77(H), CP-9(H), and MIX(H) groups at week eight (Figure 1C,D).

### 3.2. The Supplement of AP-32, bv-77, and CP-9 Improved Dyslipidemia in Obese Rats

**Figure 2:**
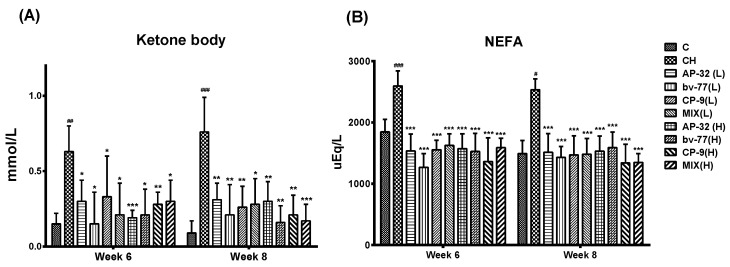
Serum levels of ketone bodies and NEFA in response to probiotic treatments. SD rats were fed a high-fat diet to induce obese symptoms from week 1 to week 8, and probiotic treatments were administered from week 5 to week 8. Obese rats were treated with a single probiotic strain or a mixture of three strains at a low or high (L = 5 × 10^9^ CFU/kg/day, H = 2.5 × 10^10^ CFU/kg/day) dosage daily for 4 weeks. The levels of (**A**) ketone bodies (mmol/L) and (**B**) NEFA (uEq/L) were presented at weeks 6 and 8. Data are shown as the mean ± S.D. Statistical analysis was performed by using a one-way ANOVA. Statistical difference was compared between the C and CH groups (^#^ *p* < 0.05, ^##^ *p* < 0.01, ^###^ *p* < 0.001), or between the CH and a probiotics-treated group (* *p* < 0.05, ** *p* < 0.01, and *** *p* < 0.001). NEFA: nonesterified fatty acids; C: control; CH: symptom control; MIX: probiotic mixture of AP-32, bv-77, and CP-9.

Excess fat was provided in the HFD, which resulted in dyslipidemia in obese rats. The blood sample of each rat was collected from the tail at five time points: before obesity induction at week 0, before probiotic intervention at weeks two and four, and after probiotic intervention at weeks six and eight. At week six, the level of ketone bodies was 0.15 ± 0.07 mM in the C group and 0.63 ± 0.17 mM in the CH group. At week eight, the level of ketone bodies was 0.09 ± 0.08 mM in the C group and 0.76 ± 0.23 mM in the CH group. The results indicated a significant elevation (^##^ *p* < 0.01, ^###^ *p* < 0.001) in response to the HFD treatment in the CH group compared to the C group (Figure 2A). The intervention of probiotic strains displayed a notable influence on the stability of ketone body levels in obese rats. Supplements of either a single probiotic strain (AP-32, bv-77, and CP-9) or a mixture of three strains at either low or high dosages significantly (* *p* < 0.05, ** *p* < 0.01, and *** *p* < 0.005) prevented the elevation of ketone bodies at weeks six and eight (Figure 2A).

Non-esterified fatty acid (NEFA) plays an important role in fatty acid metabolism, and an elevated NEFA concentration is a risk factor for cardiovascular disease [23]. At week six, NEFA levels were 847.00 ± 205.81 mg/dL in the C group and 2596.53 ± 245.63 mg/dL in the CH group. At week eight, NEFA levels were 489.79 ± 215.85 mg/dL in the C group and 2533.96 ± 177.66 mg/dL in the CH group. The HFD treatment significantly induced higher (^#^ *p* < 0.05, ^###^ *p* < 0.005) NEFA values in the CH group compared to the C group at weeks six and eight (Figure 2B). Administrations of either a single probiotic strain or the mixture of three strains significantly stabilized (*** *p* < 0.005) NEFA values during the period (Figure 2B). The HFD treatment induced a higher feed efficiency, and all probiotic groups showed similar results to the CH group (Figure S2).

The effects of probiotic intervention on blood lipids, such as cholesterol (CHOL), triglyceride (TG), low-density lipoprotein (LDL), and high-density lipoprotein (HDL), were also evaluated (Table 1 and Table 2). Compared to the C group, the HFD treatment induced higher TG and LDL as well as lower HDL at week six. Higher TG and CHOL as well as lower HDL was seen in the CH group at week eight (^#^ *p* < 0.05, ^##^ *p* < 0.01). Compared to the CH group, the supplement of bv-77(H) reduced CHOL at week six (* *p* < 0.05). The supplement of AP-32(L), MIX(L), and all probiotic groups at a high dosage reduced CHOL at week eight (* *p* < 0.05, ** *p* < 0.01). The supplement of AP-32(H), CP-9(H), and MIX(H) reduced TG at week six (* *p* < 0.05, ** *p* < 0.01). The supplement of AP-32(L), CP-9 (L), MIX(L), and all probiotic groups at the high dosage reduced TG at week eight (* *p* < 0.05, ** *p* < 0.01). The supplement of MIX(L), AP-32(H), CP-9(H), and MIX(H) reduced LDL at week six (* *p* < 0.05, *** *p* < 0.001). The supplement of AP-32(L), MIX(L), and all probiotic groups at the high dosage reduced LDL at week eight (* *p* < 0.05, ** *p* < 0.01, and *** *p* < 0.001). The supplement of CP-9(L), bv-77(L), MIX(L), and all probiotic groups at the high dosage increased HDL at weeks six and eight (* *p* < 0.05, ** *p* < 0.01).

### 3.3. The Supplement of AP-32, bv-77, and CP-9 Improved Metabolism in Obese Rats

In order to elucidate the effect of probiotic supplements on metabolism, the serum levels of glucose, aspartate aminotransferase (AST), alanine transaminase (ALT), uric acid, creatinine, sodium (Na^+^), and potassium (K^+^) were tested (Table 1 and Table 2). The glycemic level remained stable and was not affected by either the HFD treatment or probiotic treatments at week six. It is notable that the glycemic level was lower in the AP-32(H) group at week eight (* *p* < 0.05). The HFD treatment significantly elevated AST and ALT in the CH group compared to the C group at weeks six and eight (^#^ *p* < 0.05, ^##^ *p* < 0.01). The supplement of probiotics significantly reduced AST and ALT compared to the CH group at weeks six and eight (* *p* < 0.05, ** *p* < 0.01, and *** *p* < 0.001). The HFD treatment elevated uric acid and creatinine levels in the CH group compared to the C group at weeks six and eight (^##^ *p* < 0.01, ^###^ *p* < 0.001). The supplement of probiotics significantly reduced uric acid levels compared to the CH group at weeks six and eight (* *p* < 0.05, ** *p* < 0.01, and *** *p* < 0.001). The supplement of AP-32(H), bv-77(H), and MIX(L) significantly reduced creatinine at week six (* *p* < 0.05, ** *p* < 0.01). The supplement of probiotics significantly reduced creatinine at week eight (* *p* < 0.05, ** *p* < 0.01). The levels of Na^+^ and K^+^ were monitored during probiotic treatments at week six, and stable results were seen in all animals.

### 3.4. The Supplement of AP-32, bv-77, and CP-9 Reduced Fat Accumulation in Obese Rats

**Figure 3:**
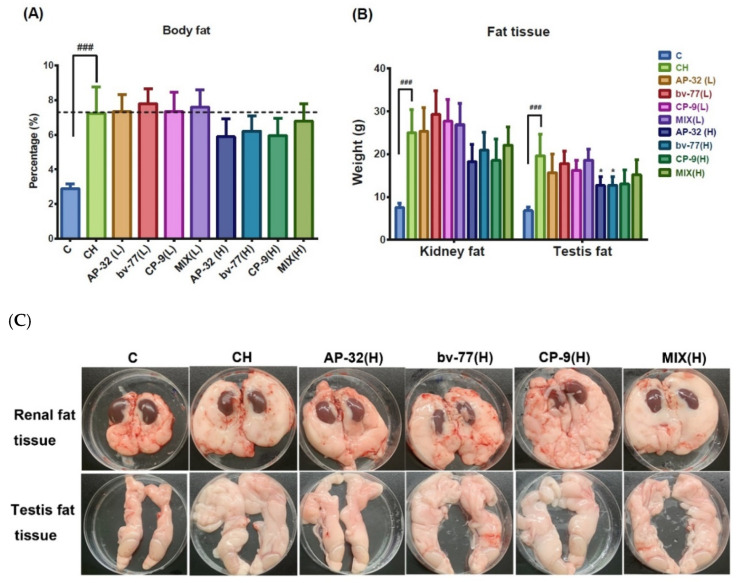
Analyses of body fat formation with and without probiotic treatments. After 4 weeks of probiotic treatments, rats were sacrificed at week 8. Fat tissues around the kidney and testicle were isolated and weighed. The bar graphs represent values of (**A**) the percentages of body fat to body weights or (**B**) net tissue weights of kidney fat and testis fat. (**C**) Photographs represent renal fat and testis fat in a regular-fed control rat (C), a high-fat diet obese rat (CH), and rats treated with a single probiotic strain or a mixture of three strains at the high dosage. Data are presented as the mean ± S.D. of six animals in each group. Statistical analysis was performed by using a one-way ANOVA. Statistical difference was compared between the C and CH groups (^###^ *p* < 0.001) or the CH and a probiotics-treated group (* *p* < 0.05). C: control; CH: symptom control; MIX: probiotic mixture of AP-32, bv-77, and CP-9; L = 5 × 10^9^ CFU/kg/day; H = 2.5 × 10^10^ CFU/kg/day.

Rats were euthanized at the end of the experiment at week eight, and the effects of the probiotic treatments on body fat formation were evaluated by visual examinations and measurements of body fat and tissue fat (Figure 3). Percentages of body fat were 2.88 ± 0.28% in the C group and 7.24 ± 1.52% in the CH group. The content of body fat was significantly higher (^###^ *p* < 0.005) in the CH group compared to the C group. High-dose supplements of probiotics showed a mild reduction in body fat increment; however, the effect was not significant compared to the CH group (Figure 3A). Fat tissues were collected from the area around the kidney and testes. The weights of the kidney fat were 7.54 ± 1.05 g in the C group and 24.96 ± 5.49 g in the CH group. The weights of the testis fat were 6.82 ± 0.80 g in the C group and 19.58 ± 5.10 g in the CH group. The net tissue weights of both areas were significantly higher (^###^ *p* < 0.005) in the CH group compared to the C group (Figure 3B). The weights of kidney fats showed a mild, but not significant, reduction in groups supplied with a high dose of probiotics. The weights of the testis fats showed significant (* *p* < 0.05) decreases in the AP-32(H) and bv-77(H) groups compared to the CH group, while CH-9(H) and MIX(H) showed mild, but not significant, reductions (Figure 3B).

The high-dose supplement of a single probiotic strain or a mixture of three strains had a more profound impact on the weights of renal and testis fat than the low-dose supplement. Fat tissues were placed in a standard 10 cm Petri dish, and the direct observation of the renal and testis fat tissues also confirmed more body fat formations in the CH group than in the C group (Figure 3C). The renal fat tissue of a CH rat was thick and solid, and covered a large area of the dish. The renal fat tissues of rats supplied with a high dose of probiotics were thinner and softer, and covered fewer areas of the dish than those of a CH rat. The testis fat tissue of a CH rat was rich and covered a larger area of the dish than that of a C rat. Testis fat tissues of rats supplied with a high dose of probiotics were more in shape and covered fewer areas than those of a CH rat, although still more than a C rat (Figure 3C).

### 3.5. The Supplement of AP-32, bv-77, and CP-9 Changed Gut Microbiota Composition in Obese Rats

**Figure 4:**
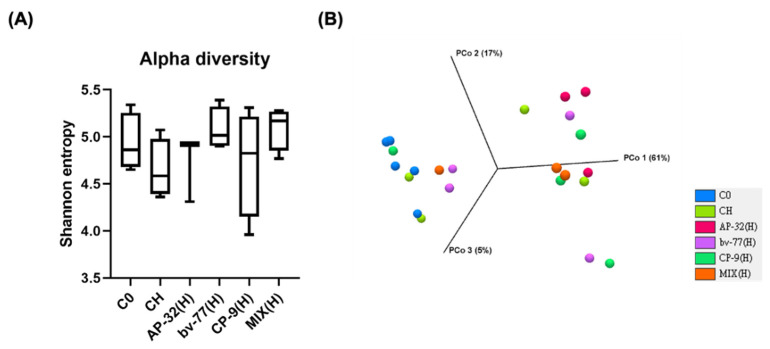
The diversity of the gut microbial composition was not significantly affected by 8-week probiotic treatments. Gut microbiota were analyzed by 16S rRNA amplicon sequencing in SD rats after probiotic treatments for 8 weeks. (**A**) The Shannon diversity index (mean ± SEM) of the microbial community was analyzed in blank control (C0), symptom control (CH), and probiotic groups. (**B**) The beta diversity of the gut microbial community was presented by the PCoA plot at the OTU level on week 8 with a weighted UniFrac distance derived from 16S rRNA sequencing data. No significant difference was seen between each group. MIX: probiotic mixture of AP-32, bv-77, and CP-9; H = 2.5 × 10^10^ CFU/kg/day.

**Figure 5:**
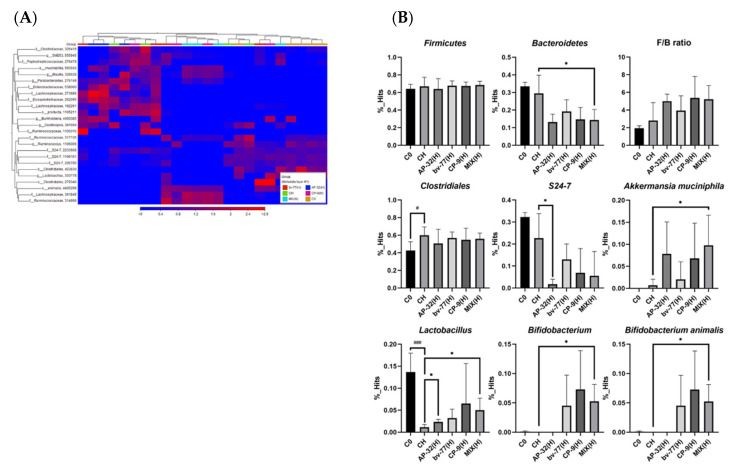
Core gut microbiota were altered by 8-week probiotic treatments. Core microbiota were determined by the heatmap analysis based on the operational taxonomic unit (OTU) tables. (**A**) Rows represent the 25 bacterial genera that were significantly enriched either in the control or probiotic-treated rats. Columns represent the 24 samples of the control and probiotic-treated rats. (**B**) Bar graphs showing relative abundance changes in core microbiota from the heatmap biplot analysis. F/B ratio: *Firmicutes*-to-*Bacteroides* ratio. Data show the mean ± SD of each group. Statistical analyses were performed by using the Student’s t-test. Statistical difference was shown as a comparison between the C0 and CH groups (^#^ *p* < 0.05 and ^###^ *p* < 0.001) or the CH and probiotic groups (* *p* < 0.05). C0: blank control; C: control; CH: symptom control; MIX: probiotic mixture of AP-32, bv-77, and CP-9; H = 2.5 × 10^10^ CFU/kg/day.

To investigate whether the impacts of the three probiotic strains on the reduction in fat accumulation resulted from gut microbiota change, total species richness and evenness (alpha diversity, Figure 4A), as well as microbial compositions between groups (beta diversity, Figure 4B), were firstly analyzed with a focus on high-dose supplementation of a single probiotic strain or a mixture of three of them. All three probiotic strains or their combination did not significantly alter the alpha and beta diversity (Figure 4). To further elucidate which core bacteria might participate in their effects on the reduction in fat accumulation in obese rats, heatmap and LEfSe analyses were used. Although the heatmap analysis shows any significant change after probiotic interventions (Figure 5A and Figure S3), the LEfSe analysis shows that *Bifidobacterium* and *A. muciniphila* were significantly increasing only after a combined supplement of three probiotic strains (Figure 5B and Figure S4, both * *p* < 0.05). Notably, the HFD-mediated decrease in *Lactobacillus spp.* was reversed upon AP-32(H) and the combined supplement of three probiotic strains (Figure 5B, both * *p* < 0.05). *Bacteroides* spp., instead, were significantly decreased only after a mixture of three probiotic strains (Figure 5B). The *Firmicutes/Bacteroides* (F/B) ratio, a well-known biomarker in obesity, did not show any significant change among each treatment group (Figure 5B). It was intriguing to know if any sub-species of *Bifidobacterium* and *Lactobacillus* spp. was changed by the treatments (Figure S5). In *Lactobacillus* spp., *L. hayakitensis* was significantly enriched by AP-32(H) and the combined supplement of three probiotic strains (Figure S5A, * *p* < 0.05 and ** *p* < 0.01), while *L. intermedius* was significantly induced only after three probiotics in combination (Figure S5A, * *p* < 0.05). Intriguingly, all *Bifidobacterium* spp. were significant increased only after treatment with the mixture of three probiotics, except *B. gallicum*, which was additionally increased by CP-9 (Figure S5B, * *p* < 0.05).

### 3.6. Functional Pathways of Energy and Retinol Metabolism Were Modulated by Probiotic Intervention

**Figure 6:**
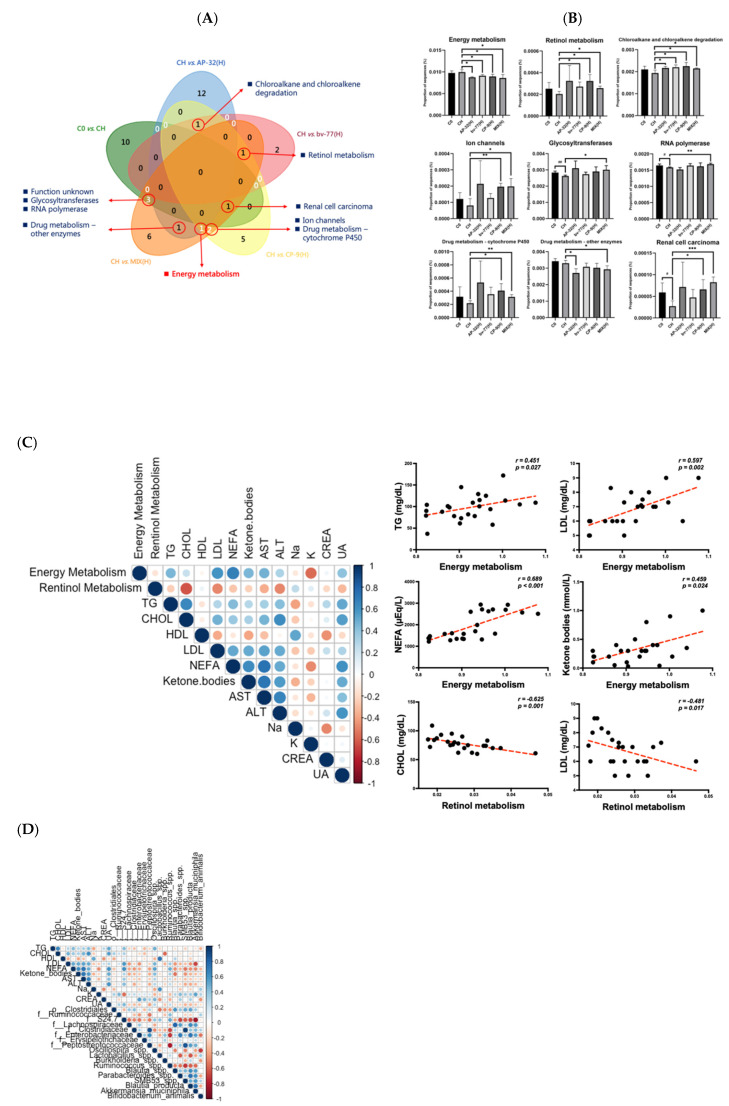

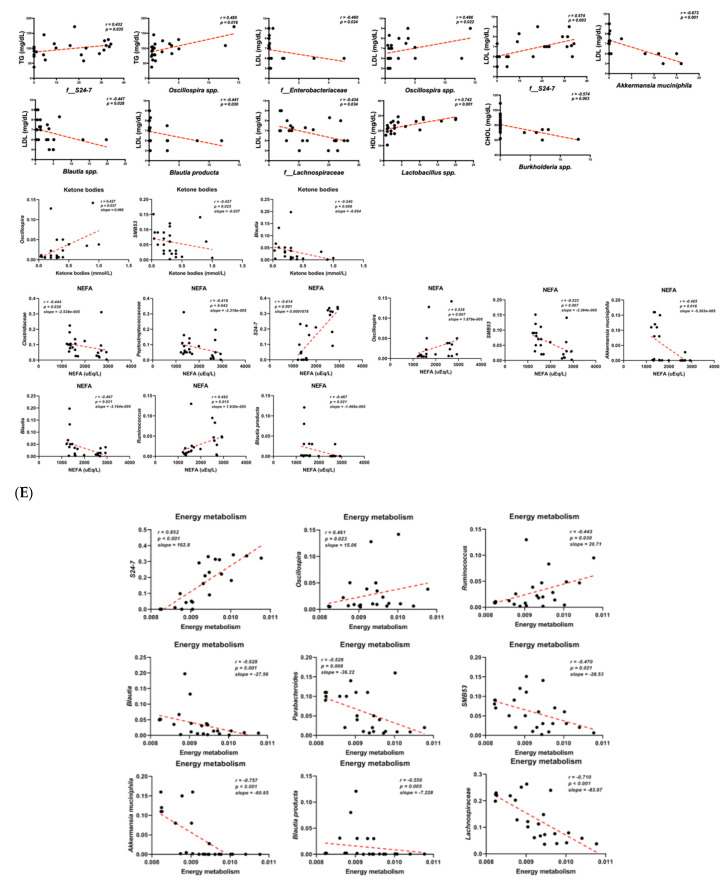
Functional pathways modulated by gut microbiota and association. A total of nine key functional pathways were found to be associated with the probiotic treatments. (**A**) Venn plot of functional pathways affected by changes in gut microbiota. (**B**) Bar graph showing the nine functional pathways responsive to the probiotic treatments. (**C**) Spearman’s correlation heatmap displayed the relationship of blood biochemistry with energy metabolism and retinol metabolism. Scatter plots displayed the energy metabolism was positively correlated with NEFA, ketone bodies, TG, and LDL. Retinol metabolism was negatively correlated with CHOL and LDL. (**D**) Spearman’s correlation heatmap displaying the relationship of blood biochemistry with gut microbiota. Scatter plots displaying that ketone bodies were positively correlated with *Oscillospira* and negatively correlated with *SMB53* and *Blautia*. NEFAs were positively correlated with *Oscillospira*, *S24-7*, and *Ruminococcus*, and negatively correlated with *SMB53, Blautia*, *Clostridaceae, Peptostreptococcaceae*, and *A. muciniphila*. (**E**) Scatter plots displaying that energy metabolism was positively correlated with *S24-7*, *Oscillospira*, and *Ruminococcus*. In constant, it was negatively correlated with *Lachnospiraceae*, *SMB53*, *Blautia*, *Parabacteroides*, and *A. muciniphila*. Statistical analyses were performed by using the Student’s t-test. Statistical difference is shown as comparison between the CH and probiotics-treated groups (^#^ *p* < 0.05, ^##^ *p* < 0.01; * *p* < 0.05, ** *p* < 0.01, *** *p* < 0.001).

A PICRUSt (Phylogenetic Investigation of Communities by Reconstruction of Unobserved States) analysis was used to figure out which metabolic/signaling pathways might be involved in the impacts of these probiotic interventions (Figure S6A–E). It is noted that gut microbiota had been mentioned as one of the confounding factors responsible for the control of body weight and energy metabolism, with a connection to obesity [24]. A total of 45 functional pathways were modulated by probiotic treatments alone or in combination as compared to the HFD control (CH, Figure 6A). The Venn diagrams illustrated the numbers of shared functional pathways across each group. The across pathway of the AP-32, CP-9, and MIX groups was energy metabolism. The across pathway of the bv-77, CP-9, and MIX groups was retinal metabolism. The across pathway of the C0, CP-9, and MIX groups was renal cell carcinoma. The across pathways of the AP-32, CP-9, and bv-77 treatment were chloroalkane and chloroalkene degradation. The across pathways of the C0 and MIX groups were the function unknown, glycosyltransferases, and RNA polymerase. Across AP-32 and MIX groups were found: drug metabolism—other enzymes. The across pathways of the CP-9 and MIX groups were the ion channel and drug metabolism—cytochrome P450 (Figure 6A,B). In the Venn plots, energy metabolism dramatically decreased in all treated groups as compared to the HFD control (Figure 6A,B). Previous reports have also revealed that retinol metabolism participates in the regulation of obesity [25,26]. Retinol metabolism increased in the bv-77, CP-9, and MIX groups (Figure 6B). Eight pathways increased upon the combined treatment with three probiotic strains, except energy metabolism, as compared with the CH group (Figure 6B). Energy and retinol metabolisms, in addition to seven other pathways, were further analyzed in regard to their potential link to obesity. Notably, the unknown pathway function was not shown here. Spearman’s correlation analysis was used to analyze the correlation between these two signaling pathways and serum lipid profiles (Figure 6C). Energy metabolism was positively correlated with serum levels of TG, LDL, NEFA, and ketone bodies. Retinal metabolism was negatively correlated with serum CHOL and LDL levels (Figure 6C). These results suggested that probiotic strains might decrease serum levels of certain lipid-related characteristics through negatively regulating energy metabolism but positively regulating retinol metabolism. Furthermore, serum TG levels were positively correlated with *Oscillospira* and *f__S24-7.* LDL levels were positively correlated with *Oscillospira* and *f__S24-7*, but negatively correlated with *Blautia producta*, *A. muciniphila, f__Enterobacteriaceae*, and *f__Lachnospiraceae*. CHOL levels were negatively correlated with *Burkholderia* spp. HDL levels were positively correlated with *Lactobacillus* spp. Ketone bodies were positively correlated with *Oscillospira*, but negatively correlated with *SMB53* and *Blautia* spp. NEFA levels were positively correlated with *Oscillospira, f__S24-7*, and *Ruminococcus*, but negatively correlated with *A. muciniphila, Blautia producta, SMB53, f__Clostridiaceae*, and *f__Peptostreptococcaceae* (Figure 6D). These results showed the link of the abundance of core bacteria of the gut to blood lipid profiles, suggesting that alterations in the microbial abundance might contribute to metabolic outcomes. Accordingly, we found that energy metabolism was positively correlated with *Oscillospira, Ruminococcus*, and *f__S24-7*, but negatively correlated with *Blautia producta*, *A. muciniphila, Parabacteroides, SMB53*, and *f__Lachnospiraceae* (Figure 6E). The results presented the correlation of the abundance of single-core bacteria of the gut with metabolic function pathways, indicating that alterations in the abundance of the gut bacteria mediated by probiotic treatments might change metabolic ability.

In short, a combined supplement of three probiotic strains significantly increased the abundance of *Akkermansia mucinphila* but decreased energy metabolism compared with obese (CH) rats. The abundances of energy-metabolism-related gut bacteria were positively correlated with serum TG, LDL, NEFA, and ketone body levels, while the abundance of *Akkermansia mucinphila* was negatively correlated with serum LDL and NEFA levels. Additionally, a negative correlation of the abundance of *Akkermansia mucinphila* with energy metabolism was observed. Therefore, a combined supplement of three probiotic strains could increase the abundance of *Akkermansia mucinphila* to reduce serum NEFA levels potentially mediated through its negative regulation of energy metabolism.

## 4. Discussion

Due to the close correlation of obesity with many other metabolic diseases and health problems, numerous efforts and products have been invested to solve, or at least control, excess fat accumulation in the body. It was not until recently that the important role of gut microbiota in obesity has come to light [27,28]. In our study, the effect of probiotic strains, AP-32, bv-77, and CP-9, on alleviating body fat formation was demonstrated in an animal model of obesity. In general, most of the results showed dose-dependent effects, and results from our low-dose administrations of probiotics hardly claimed a stable influence. The high-fat diet (HFD) induced obesity in 4 weeks; some effects of the probiotic strain were seen in 2 weeks, while others were not seen until later, in 4 weeks. The result indicated that different probiotic strains may require different treatment periods to reach significant bio-impacts.

*Lactobacillus salivarius* subsp. *Salicinius* AP-32 was reported to modulate the inflammatory responses of epithelial and immune cells in vitro [29], and showed the potential to eradicate *Helicobacter pylori* infection in vivo [30]. Heat-killed AP-32 was reported to skew the immune response toward Th1 polarization and enhanced immunomodulatory potential [31]. Both viable and heat-killed AP-32 had antibacterial activity against oral pathogens [32]. In this study, AP-32 displayed an impact on the control of body weight at both low and high dosages, as well as a high potential for the control of blood sugar. These results were in good agreement with previous studies on diabetic control. AP-32 decreased glycemic levels, reduced β-cell death, and attenuated diabetes-mediated liver and kidney injury in animal models [33,34]. Therefore, AP-32 may be classified into a population of gut microbiota, which plays a role in balancing dietary fat and glucose metabolism in the human body [35]. *Lactobacillus rhamnosus* bv-77 displayed an influence on reducing plasma cholesterol levels sooner than the other two strains (Figure 5B). Hypercholesterolemia is a major risk factor for cardiovascular disease, and some probiotic strains are reported to have cholesterol-lowering capabilities via the deconjugation of bile acids or the modulation of intestinal cholesterol uptake and hepatic cholesterol efflux [36,37]. In our study, bv-77 prevented OA-induced TG accumulation in an intestinal cell model and elucidated its capacity in cholesterol-lowering in the animal model. However, the mechanism behind this phenomenon has not yet been pursued. Further studies are required to reveal the relationship of bv-77 and cholesterol metabolism in the human body. Studies have shown health benefits of *Bifidobacterium animalis* subsp. *lactis* strains for the human body in many aspects [38,39]. The CP-9 strain was reported to decrease glycemic levels and reduce β-cell death in an animal model [34]. Both viable and heat-killed CP-9 had antibacterial activity against oral pathogens [32]. The blood glucose level was elevated in animals treated with CP-9, but the difference did not reach statistical significance. In a previous study, *B. animalis* CP-9 and *L. rhamnosus* bv-77 displayed weak capability in sugar consumption in vitro. Only the reducing effect of *L. salivarius* AP-32 on the glycemic level was validated in vivo [33]. In this study, the capability of *L. salivarius* AP-32, *B. animalis* CP-9, and *L. rhamnosus* bv-77 in reducing TG accumulation was first analyzed in vitro (Figure S1). The effect of *B. animalis* CP-9 on reducing body weight, ketone bodies, NEFA, cholesterol, TG, and LDL was validated in vivo. Results from these two studies indicated that the *B. animalis* CP-9 strain may play a more significant role in modulating lipid metabolism than glucose metabolism in the host. The depletion of *B. animalis* species was reported to be associated with obesity [40], and the supplement of *B. animalis* CP-9 was able to increase the population of this species in the gut (Figure S5). Obesity is a multifactorial chronic disease and often results in metabolic syndrome, which is a combination of multiple medical conditions. The probiotic supplement containing multispecies may compensate for the weakness of monostrains, exert synergistic effects, and effectively reverse microbial dysbiosis. On the other hand, CP-9 displayed an impact on reducing the plasma LDL level sooner than the other two strains (Figure 6D). LDL is involved in atherosclerosis and high LDL induces inflammation, which is associated with an increased risk of cardiovascular diseases [41]. CP-9 showed good potential on balancing lipoproteins, and further studies are needed to elucidate the mechanism underlying the LDL-lowering effect.

Different probiotic strains show synergistic effects and function better in combination [42]. The mixture of AP-32, bv-77, and CP-9 displayed an influence on reducing plasma LDL and serum creatinine levels at a lower dosage than single strains (Figure 6B). Although the mixture of the three probiotic strains did not provide a strong synergistic effect on reducing weight gain and body fat formation, it balanced the beneficial effect of the three strains. Supplements of either single strains or the mixture reduced ketone body levels without altering feed efficiency (Figure 3). This result indicated that these strains of bacteria may consume lipids in the gut and reduce lipids available for intestinal absorption. The HFD reduced HDL (AKA, the good cholesterol) and increased other blood lipids, such as NEFA, cholesterol, triacylglycerol, and LDL (Figure 2). The elevated blood lipids were lowered by administrations of AP-32, bv-77, and CP-9, and the effect was dose-dependent. In general, treatments at the high dosage displayed the effect earlier, at week six, and treatments at the low dosage displayed the effect later, at week eight. These results suggest that supplements of AP-32, bv-77, and CP-9 were beneficial to prevent or manage coronary heart diseases [43].

The liver is thought to be the most complicated organ and responsible for up to 500 functions. Liver cells break down fats and produce energy in fat metabolism, and excess fat intake causes liver damage in obese rats, resulting in higher levels of AST and ALT (Table 1 and Table 2). The maintenance of liver function forms a positive cycle on stabilizing fat metabolism and reducing fat accumulation [42]. Some strains of *Lactobacillus* have been shown to reduce TG content in the liver and alleviate inflammation of alcoholic steatohepatitis in a mouse model [44]. In this study, probiotic strains AP-32, bv-77, and CP-9 reduced serum AST and ALT levels, indicating a relief on fat metabolism and the restoration of liver function. Obese adipose tissue plays a pivotal role in the development of insulin resistance [45]. In the present study, serum ALT and AST levels were increased in CH rats, revealing dysfunction or damage of the liver in obese (CH) animals. It is notable that the liver is regarded as the most sensitive organ undergoing insulin impairment compared to other organs [45]. Long-term, low-grade inflammation and elevated NEFA prevent insulin from carrying out its action in the liver [46,47]. Therefore, the liver damage may further result in hepatic insulin resistance if the HFD feeding lasted longer. Notably, the blood glucose levels did not significantly increase in our study. Probiotic treatments reduced serum NEFA, ALT, and AST to levels comparable to normal (C) animals. The protective effect of probiotic treatment in the liver may come from the modulation of gut microbiota. The HFD caused the dysbiosis of gut microbiota and resulted in abnormal energy metabolism. When excess fat is deposited over the liver, the onset of hepatic insulin resistance and then systemic insulin resistance is expected. The supplement of probiotics restored microbiota homeostasis and energy metabolism. When lipid uptake was reduced in adipose tissue, hepatic lipotoxicity was alleviated and insulin resistance was prevented. In individuals affected by obesity, a compensatory hyperfiltration occurs to meet the heightened metabolic demands of increased body weight. The increase in intraglomerular pressure can damage the kidneys and raise the risk of developing chronic kidney diseases in the long term [48]. In this study, the function of the kidney was disturbed by the HFD, resulting in higher levels of uric acid and creatinine (Table 1 and Table 2). Probiotic strains AP-32, bv-77, and CP-9 stabilized uric acid and creatinine levels, indicating the potential on lowering the risk of gout, chronic kidney diseases, and diabetes, which are closely associated with obesity [49]. Electrolytes in blood are not affected by either obese symptom or probiotic administrations. Therefore, supplements of these probiotic strains were safe in patients with electrolyte abnormality.

The in vitro culture of *Akkermansia muciniphila* (*A. muciniphila*) was difficult in general, and, notably, this bacterium could be enriched by our combined probiotic treatment. *A. muciniphila* is a beneficial microbe that colonizes in the human intestine, and has drawn much attention due to its inverse correlation with obesity. The abundance of *A. muciniphila* decreased in obese mice and the elevation of this bacterium could reverse the inflammation, insulin resistance, and metabolic heart diseases induced by a Western diet [50]. It is also inversely associated with metabolic disorders, such as obesity, low-grade inflammation, diabetes, and cardiovascular diseases [50]. Unfortunately, the in vitro incubation of *A. muciniphila* requires stringent culture conditions and a specific animal-based medium (i.e., mucin from an animal source). Although it may survive under microaerophilic conditions, this bacterium is very sensitive to oxygen [51]. These demands challenge the manufacturing of *A. muciniphila*, limit the evaluation of its potential for humans, and obstruct its therapeutic perspectives. Our results showed that a combined supplement of three strains, AP-32, bv-77, and CP-9, significantly increased the abundance of *A. muciniphila*, which was inversely correlated with serum NEFA and LDL levels, in addition to the predictive energy metabolism pathway. This exciting result provides the basis of the potential application of a mixture of these three probiotic strains in weight and obesity control. Although the manufacturing of living *A. muciniphila* is still under development, our results may provide another indirect or direct strategy for developing novel food or pharma supplements with the beneficial effects of *A. muciniphila*.

*Oscillospira* is an anaerobic bacterial genus from the *Clostridial* cluster, and some species of them have been found to have the ability to utilize glucoronate [52]. In this study, the abundance of *Oscillospira* was positively correlated with the levels of NEFAs, TG, and LDL, and was reduced by a mixture of three strains in rats. However, the abundance of *Oscillospira* was reported to be inversely correlated with serum TG levels in humans, and a low abundance of *Oscillospira* was found in hypertriglyceridemia patients [53,54]. This controversial result implicates the difference of energy metabolism between rodents and primates. It is noted that *Oscillospira* can be enriched by a HFD, but hardly increased by a high-glucose diet in rats [55]. The growth of *Oscillospira* requires specific carbon sources, and glucose in particular, significantly promotes its growth [56]. Moreover, there are many different species and strains of *Oscillospira*, but their specific roles in human health are still far from clear [57]. Notably, a similar result was also observed in the case of S24-7. *S24-7* is newly named *Muribaculaceae*, and belongs to a family of bacteria within the order *Bacteroidales* [58]. This commensal bacterium is one of major mucin monosaccharide foragers in the gut, which makes it an attractive candidate for therapeutic interventions to compete with pathogens for such nutrients [59]. Based on this nature, *S24-7* is thought to be involved in carbohydrate metabolism in humans [60]. In the present study, the abundance of *S24-7* was also positively correlated with the levels of NEFAs, TG, and LDL in rats. However, the abundance of *S24-7* was reported to be decreased in HFD-treated Streptozotocin-induced type two diabetic mice [61]. Although much evidence has linked changes in gut microbiota to obesity, controversial results obtained in this field have shaded the existence of suitable bacteria as a biomarker [62]. More studies are needed to understand whether these bacteria are involved differently in glucose and fat metabolism.

Obesity is one of very first warning signs for unhealthy physical conditions, commonly caused by excessive food intake and insufficient exercise. The imbalance in the composition of the microbiota can cause obesity, but not necessarily be the origin of it [63]. In fact, it is usually the consequence of sedentary lifestyles, and, in turn, affects the homeostasis of energy metabolism. Supplements of functional probiotics are able to restore the gut flora, but earlier colonizers of a species can inhibit colonization by a naïve population of that species due to metabolic exclusion or slight mutational advantages [64]. It has been shown that people with extra belly fat lost 8.2–8.5% of their belly fat after drinking fermented milk for 12 weeks. Interestingly, when they stopped drinking the milk, all of their belly fat returned [65]. This study has demonstrated the conducive effects of probiotic strains AP-32, bv-77, and CP-9 on health, and better improvements in body fat formation can be expected with higher dosages and longer periods of probiotic supplements.

## Figures and Tables

**Table 1:** Analyses of blood chemistry during probiotic intervention at week 6.

Week 6	C	CH	AP-32(L)	bv-77(L)	CP-9(L)	MIX(L)	AP-32(H)	bv-77(H)	CP-9(H)	MIX(H)
Glucose (mg/dL)	102.4 ± 4.16	109.5 ± 6.35	100.8 ± 9.65	111.4 ± 5.13	116.4 ± 1.67	113.6 ± 7.86	109.2 ± 5.72	116.4 ± 6.02	115.6 ± 4.93	106.8 ± 4.15
Cholesterol (mg/dL)	80.50 ± 8.46	85.67 ± 12.52	71.00 ± 24.49	70.50 ± 18.28	89.33 ± 15.64	85.50 ± 19.04	73.00 ± 8.27	63.50 * ± 12.14	77.33 ± 13.16	71.33 ± 9.69
Triacylglycerol (mg/dL)	99.50 ± 9.05	122.50 ^#^ ± 18.36	99.00 ± 25.97	91.83 ± 37.72	101.50 ± 32.38	105.80 ± 22.29	96.00 * ± 17.10	89.67 ± 32.16	91.17 ** ± 15.52	80.67 ** ± 19.34
LDL (mg/dL)	6.63 ± 0.75	8.52 ^#^ ± 0.61	7.90 ± 0.67	7.55 ± 1.30	7.30 ± 1.22	7.22 * ± 1.10	6.90 * ± 1.19	7.72 ± 0.32	6.00 *** ± 0.18	6.42 *** ± 0.49
HDL (mg/dL)	25.28 ± 2.35	15.82 ^##^ ± 2.55	19.53 ± 5.32	21.33 * ± 4.77	21.75 * ± 4.97	23.63 ** ± 4.50	21.63 ** ± 3.41	22.83 ** ± 4.77	22.00 ** ± 2.26	20.48 * ± 3.42
AST (U/L)	103.83 ± 16.64	142.33 ^#^ ± 29.51	112.33 * ± 12.32	128.40 * ± 15.01	110.50 * ± 10.99	110.00 ** ± 5.01	107.67 ** ± 7.80	116.33 ** ± 4.27	109.80 ** ± 7.98	99.83 ** ± 13.36
ALT (U/L)	29.83 ± 5.56	40.83 ^##^ ± 4.17	29.00 ** ± 6.10	22.00 ** ± 3.58	29.00 *** ± 6.99	25.67 *** ± 1.86	30.17 *** ± 2.04	21.17 *** ± 5.60	24.40 *** ± 3.65	25.00 *** ± 3.74
Uric acid (mg/dL)	1.10 ± 0.24	2.08 ^###^ ± 0.21	1.52 ** ± 0.26	1.62 * ± 0.30	1.67 ** ± 0.10	1.55 * ± 0.30	1.35 *** ± 0.27	1.60 ** ± 0.30	1.80 * ± 0.23	1.55 ** ± 0.32
Creatinine (mg/dL)	0.25 ± 0.03	0.31 ^#^ ± 0.04	0.26 ± 0.05	0.30 ± 0.07	0.32 ± 0.03	0.25 ** ± 0.02	0.25 * ± 0.03	0.24 ** ± 0.03	0.27 ± 0.02	0.26 ± 0.03

Data are presented as the mean ± S.D. of six animals in each group. Statistical analysis was performed by using a one-way ANOVA. Statistical difference was compared between the C and CH groups (^#^ *p* < 0.05, ^##^ *p* < 0.01, and ^###^ *p* < 0.001) or the CH and a probiotics-treated group (* *p* < 0.05, ** *p* < 0.01, and *** *p* < 0.001). C: control; CH: symptom control; MIX: probiotic mixture of AP-32, bv-77, and CP-9; L = 5 × 10^9^ CFU/kg/day; H = 2.5 × 10^10^ CFU/kg/day; LDL: low-density lipoprotein; HDL: high-density lipoprotein; ALT: alanine aminotransferase, AST: aspartate Transaminase.

**Table 2:** Analyses of blood chemistry during probiotic intervention at week 8.

Week 8	C	CH	AP-32(L)	bv-77(L)	CP-9(L)	MIX(L)	AP-32(H)	bv-77(H)	CP-9(H)	MIX(H)
Glucose (mg/dL)	105.6 ± 6.47	108.75 ± 5.91	106.6 ± 5.41	105.00 ± 5.96	116.6 ± 10.92	109.2 ± 6.30	99.2 * ± 4.44	116.6 ± 6.06	107.6 ± 4.62	107.2 ± 6.02
Cholesterol (mg/dL)	79.33 ± 6.12	94.50 ^#^ ± 10.43	77.67 * ± 10.48	75.67 ± 21.81	79.50 ± 15.18	72.17 * ± 15.87	71.83 ** ± 10.68	64.17 * ± 16.07	71.33 ** ± 7.09	75.00 ** ± 7.48
Triacylglycerol (mg/dL)	98.33 ± 15.16	129.83 ^#^ ± 22.60	89.33 * ± 22.47	104.42 ± 16.82	101.00 * ± 21.56	89.17 ** ± 18.39	82.50 ** ± 11.79	81.83 * ± 29.60	89.75 * ± 37.21	80.33 ** ± 18.68
LDL (mg/dL)	6.90 ± 1.19	8.13 ± 0.80	7.60 ± 0.89	7.63 ± 1.71	6.87 * ± 0.75	6.10 *** ± 0.68	6.52 * ± 1.26	6.72 * ± 0.92	6.22 *** ± 0.83	6.00 ** ± 0.89
HDL (mg/dL)	25.60 ± 2.30	16.22 ^##^ ± 4.08	19.47 ± 4.26	24.57 ** ± 2.86	24.63 ** ± 3.24	25.77 ** ± 3.59	22.50 * ± 3.14	23.07 ** ± 2.93	23.62 ** ± 3.47	25.18 ** ± 2.69
AST (U/L)	118.80 ± 25.06	137.17 ± 19.65	105.00 ** ± 10.92	109.20 ** ± 16.58	107.83 * ± 17.52	84.67 *** ± 7.79	102.33 ** ± 9.33	88.00 *** ± 6.42	91.50 *** ± 10.01	89.83 *** ± 8.23
ALT (U/L)	33.33 ± 7.03	43.33 ^#^ ± 7.50	25.50 *** ± 4.09	22.83 *** ± 3.92	27.00 ** ± 5.40	28.00 ** ± 3.85	29.17 ** ± 4.58	25.83 *** ± 3.31	25.00 *** ± 5.55	28.83 ** ± 5.19
Uric acid (mg/dL)	1.37 ± 0.61	2.32 ^##^ ± 0.32	1.38 *** ± 0.32	1.60 * ± 0.57	1.63 *** ± 0.18	1.77 * ± 0.47	1.42 ** ± 0.40	1.67 * ± 0.55	1.38 *** ± 0.30	1.63 ** ± 0.41
Creatinine (mg/dL)	0.31 ± 0.02	0.35 ± 0.07	0.25 * ± 0.02	0.26 * ± 0.04	0.27 * ± 0.05	0.26 * ± 0.04	0.26 * ± 0.02	0.24 ** ± 0.02	0.22 ** ± 0.04	0.24 ** ± 0.04
Na^+^ (mmol/L)	151.17 ± 1.72	151.17 ± 1.94	151.33 ± 1.37	153.17 ± 0.98	150.80 ± 1.64	153.33 ± 1.21	151.50 ± 1.64	152.50 ± 2.88	152.50 ± 0.58	151.40 ± 1.14
K^+^ (mmol/L)	6.08 ± 0.26	6.20 ± 0.48	6.45 ± 0.52	6.45 ± 0.51	6.77 ± 0.48	6.38 ± 0.24	6.70 ± 0.70	6.27 ± 0.41	6.68 ± 0.90	6.55 ± 0.63

Data are presented as the mean ± S.D. of six animals in each group. Statistical analysis was performed by using a one-way ANOVA. Statistical difference was compared between the C and CH groups (^#^ *p* < 0.05, ^##^ *p* < 0.01) or the CH and a probiotics-treated group (* *p* < 0.05, ** *p* < 0.01, and *** *p* < 0.001). C: control; CH: symptom control; MIX: probiotic mixture of AP-32, bv-77, and CP-9; L = 5 × 10^9^ CFU/kg/day; H = 2.5 × 10^10^ CFU/kg/day; LDL: low-density lipoprotein; HDL: high-density lipoprotein; ALT: alanine aminotransferase, AST: aspartate Transaminase.

## Data Availability

The data presented in this study are available on request from the corresponding author.

## Supplementary Materials

The following supporting information can be downloaded at: https://www.mdpi.com/article/10.3390/nu14030527/s1, Figure S1: *L. salivarius* AP-32, *L. rhamnosus* bv-77, and *B. animalis* CP-9 reduced oleic acid (OA)-induced intestinal TG accumulation in vitro. Figure S2: HFD treatment changed feed efficiency in rats. Figure S3: Heatmap analysis of core gut microbiota after 8-week probiotic treatment. Figure S4: Core gut microbiota changed after 8-week probiotic treatment. Figure S5: *Lactobacillus* and *Bifidobacterium* were significantly increased after 8-week probiotic treatment. Figure S6: Functional pathways analysis after 8-week probiotic treatment by a PICRUSt analysis.
