# Supplementary material for: A Combined Supplement of Probiotic Strains AP-32, bv-77, and CP-9 Increased Akkermansia mucinphila and Reduced Non-Esterified Fatty Acids and Energy Metabolism in HFD-Induced Obese Rats
Source: Nutrients. 2022 Jan 26;14(3):527. doi: 10.3390/nu14030527 (PMC8839477; doi:10.3390/nu14030527)
Supplement: Supplementary file 1 [file nutrients-14-00527-s001.zip › nutrients-1542298-supplementary.pdf]

Supplementary Material

1.1. Fatty acid accumulation in cultured intestinal cells

To test whether the fatty acid consumption capacity of probiotic strains affects cellular fat accumulation *in vitro*, Caco-2 cells (BCRC60182) were co-cultured with probiotic strains using a 0.4 μm pore insert to exclude direct interactions between cells and bacteria. Briefly, Caco-2 cells were cultured at 37 °C under 5% CO_2 in (Dulbecco's Modified Eagle Medium) DMEM supplemented with 10% Fetal Bovine Serum (FBS). For experimentation, the cells were plated at a density of 2×10^5 cells per well into six-well plates and were grown for 7 days in culture medium. The experimental medium was prepared as follows: 100 μl of probiotic strain culture, grown to a concentration of 2×10^8 cfu/ml in de Man Rogosa and Sharpe (MRS) broth, was added to 10 ml of DMEM containing 500 μmol/l OA (Sigma-Aldrich, St. Louis, MO, USA), and the pH was adjusted to 7.4. Approximately 2×10^8 cfu/ml probiotic strain was seeded on a Transwell membrane (SPL, Pochon, Korea) and inserted into a six-well culture plate containing Caco-2 cells. As a no-OA (Oleic acid) control group, solely DMEM was added to the Transwell without probiotic strain seeding. As a no-probiotic control, DMEM containing OA was added to the Transwell without probiotic strain seeding. After 6 h, Caco-2 cells co-cultured with probiotic strains under OA-treated conditions were collected, and TG (triglyceride) extraction and quantification were performed according to the manufacturer's protocol (Cayman Chemical, Ann Arbor, MI, USA).

1.2. Evaluation of feed efficiency

The animal body weight was measured every 2 weeks. The total and remaining fodder was weighed as food intake every 24 hours. Feed efficiency = (weight gain ÷ food intake) × 100%.

2. Supplementary Results

2.1. Three probiotic strains, AP-32, bv-77, and CP-9, reduced TG formation in vitro

Oleic acid (OA) is a common dietary unsaturated fatty acid in human diets, and leads to triglyceride (TG) accumulation in intestinal cells. Direct interaction of cells and bacteria was prevented by using an indirect co-culture system (Figure. S1A). The endogenous TG content was 78.2±5.6% in no-OA treated Caco-2 control, and total TG content was 100±2.2% in OA treated Caco-2 control. The supplementation of OA induced a significant TG accumulation in Caco-2 cells ($^{###}p$<0.001, Figure S1B). The total TG contents were 84.4±2.4%, 85.3±0.6%, and 88.6±0.5% in AP-32, CP-9, and bv-77 treated Caco-2 cells, respectively. The supplementation of these 3 probiotic strains significantly reduced the TG accumulation in Caco-2 cells comparing to no probiotic treated Caco-2 control (****p*<0.001, Fig 1B). The total TG content was 99.0±1.8% in *Lactobacillus rhamnosus* GG (LGG) treated Caco-2 cells, and displayed no significant difference with no probiotic treated Caco-2 control.

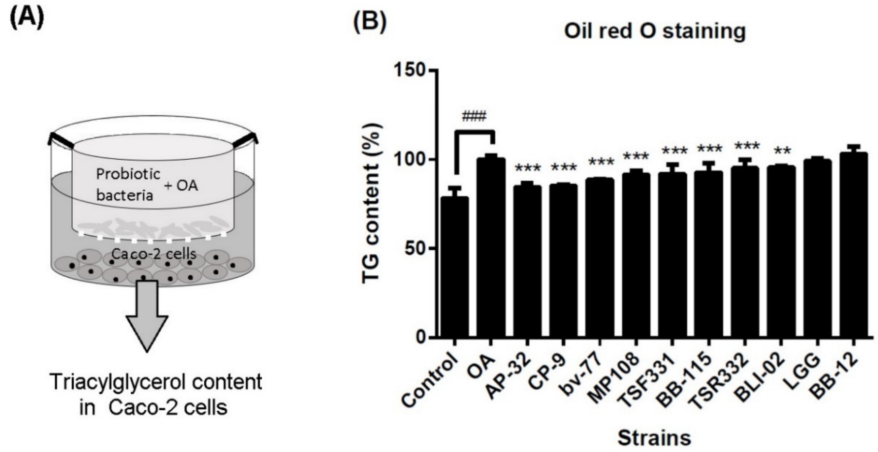

Figure S1. *L. salivarius* AP-32, *L. rhamnosus* bv-77, and *B. animalis* CP-9 reduced oleic acid (OA)-induced intestinal TG accumulation *in vitro*. (**A**) Oil Red O staining of OA-induced lipid accumulation in CaCo-2 cells co-cultured indirectly with probiotic stains. (**B**) OA-induced TG accumulation by the control and probiotic strains. Data are expressed as the mean ± SD from three independent experiments. Statistical comparisons obtained by the Student's t-test, $^{###}p<0.001$, $^{**}p < 0.01$ and $^{***}p<$ 0.001. OA: oleic acid; TG: triacylglycerol; AP-32: *Lactobacillus salivarius* AP-32; CP-9: *Bifidobacterium animalis* CP-9; bv-77: *Lactobacillus rhamnosus* bv-77; MP108: *Lactobacillus rhamnosus* MP108; TSF331: *Lactobacillus fermentum* TSF331; BB-115: *Bifidobacterium animalis* BB-115; TSR332: *Lactobacillus reuteri* TSR332; BLI-02: *Bifidobacterium longum* BLI-02; LGG: *Lactobacillus rhamnosus* GG; BB-12: *Bifidobacterium animalis* BB-12.

2.2. The supplement of AP-32, bv-77, and CP-9 did not change feed efficiency in obese rats

The feed efficiencies were recorded every 2 weeks from week 1 to week 8, and all groups showed a similar dynamic change during eight weeks. Comparing to C group, high-fat diet (HFD) treatment induced a significantly ($^{###}p<0.001$) higher feed efficiency in CH group. Feed efficiencies were not affected by probiotic intervention and all probiotic groups showed similar results to CH group.

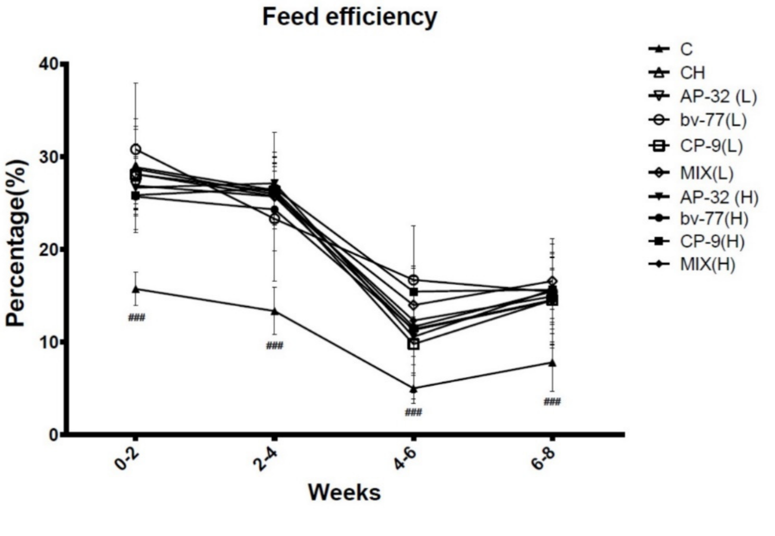

Figure S2. HFD treatment changed feed efficiency in rat. Body weight was measured every 2 weeks, and food intake was recorded every 24 hours. The statistical analysis was performed by using one-way ANOVA. The statistical difference was compared between C and CH group ($^{###}p<0.005$), or between CH and a probiotics-treated group.

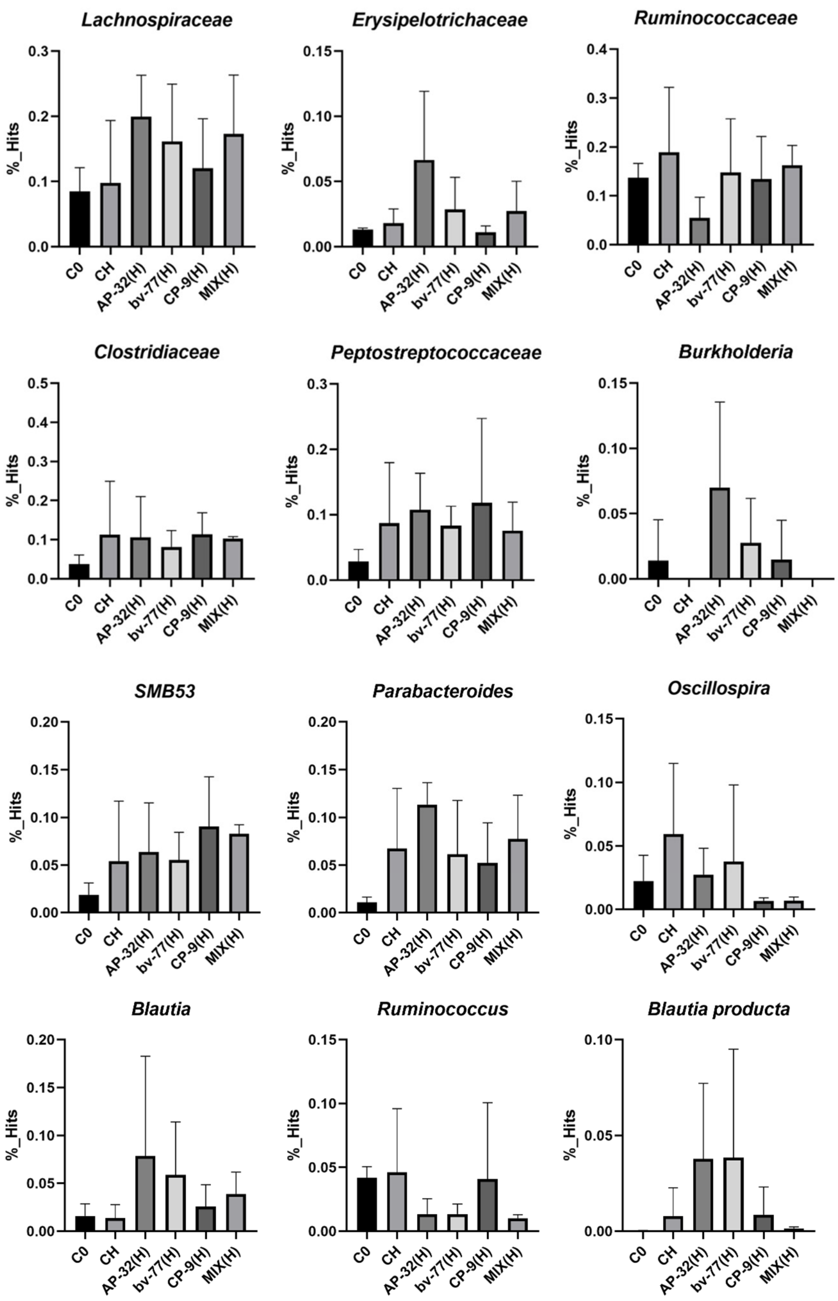

Figure S3. Heatmap analysis of core gut microbial alterations by 8-week probiotic treatments. According to Heatmap analysis results, the results did not show any significant change after probiotic treatment.

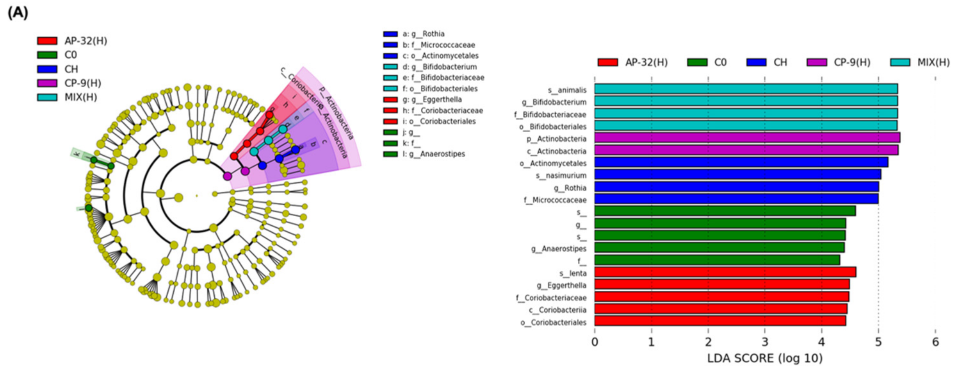

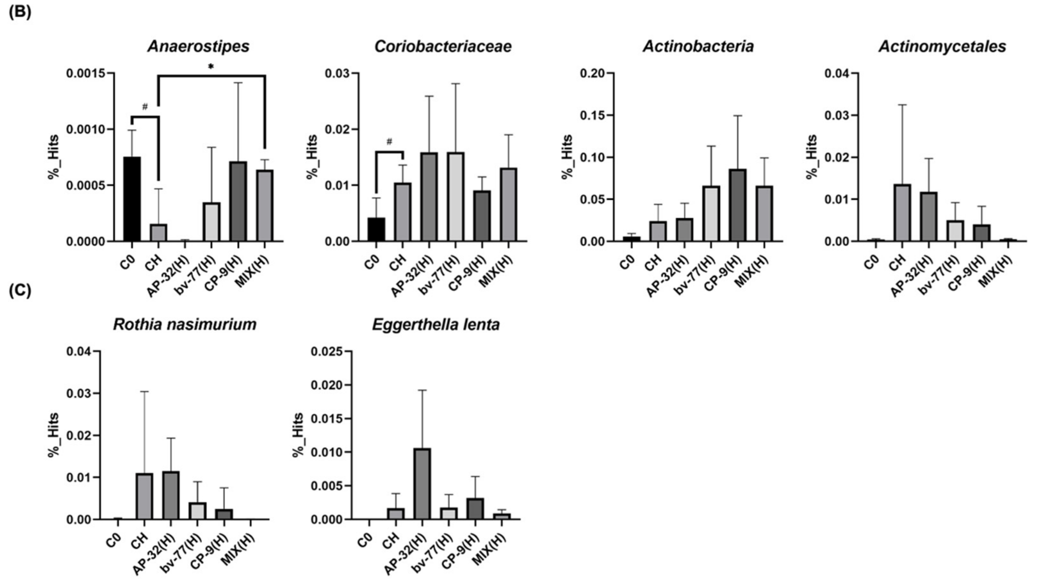

Figure S4. Core gut microbiota changed after 8-week probiotic treatment. (**A**) After 8-week probiotic treatment, core microbiota analysis by the LEfSe analysis. (**B** & **C**) Core microbiota in genus and species level were analyzed and showed as bar plot. *Anaerostipe spp.* was significantly increased compared to CH (HFD) control. The statistical analyses were performed by using the Student's t-test. Statistical difference is showed as comparison between C0 and CH group ($^{\#}p$<0.05, $^{\#\#}p$<0.01, $^{\#\#\#}p$<0.001), or CH and probiotics-treated group (*p<0.05, **p<0.01, ***p<0.001).

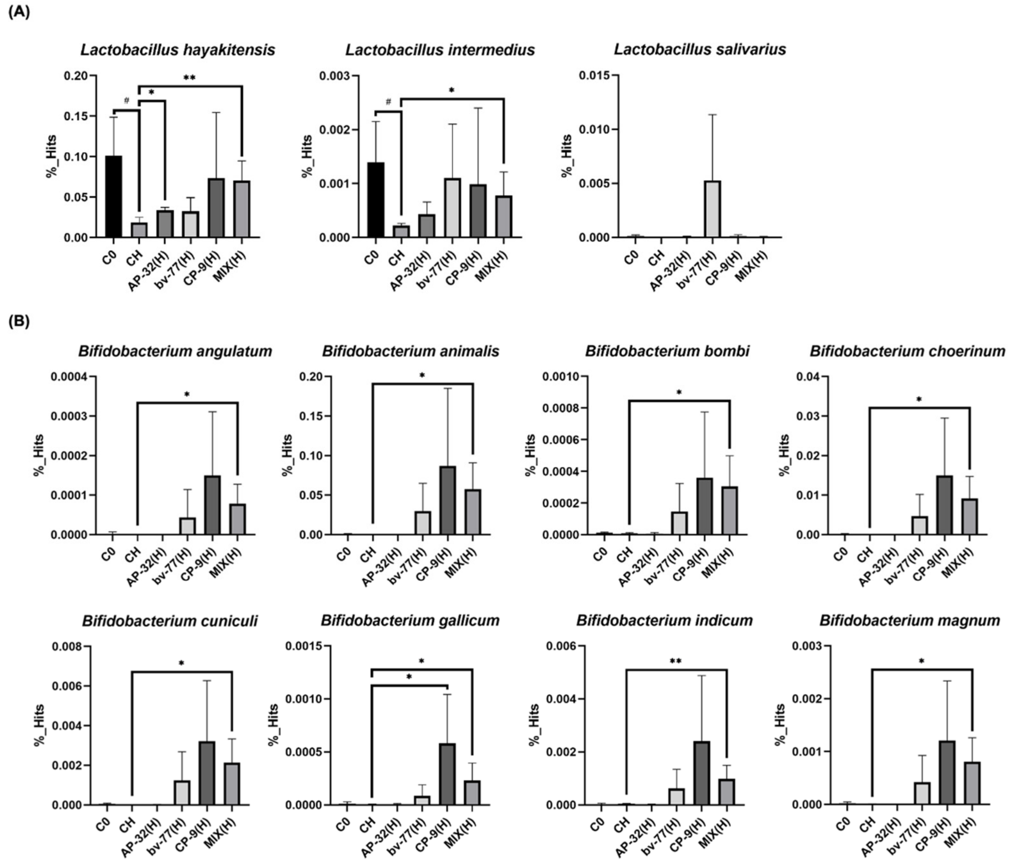

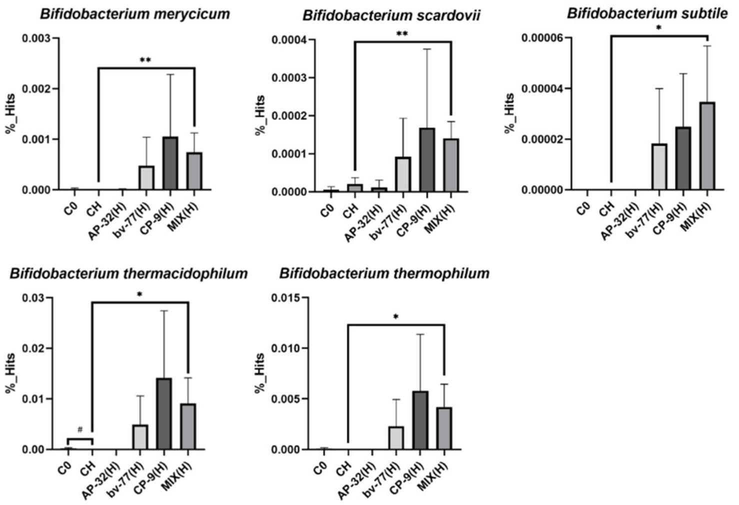

Figure S5. ***L****actobacillus* and *Bifidobacterium* were significantly increased after 8-week probiotic treatment. (**A**) *Lactobacillus hayakitensis* and *Lactobacillus intermedius* were significantly increased after 8-week combined treatment of 3 probiotic strains (MIX(H)) as compared to high-fat diet treated control (CH). (**B**) All *Bifidobacterium* in species level were significantly increased after 8-week combined treatment of 3 probiotic strains (MIX(H)). The data showed the mean±SD of each group. The statistical analyses were performed by using Student's t-test. Statistical difference is showed as comparison between C0 and CH group ($^{\#}p<0.05$, $^{\#\#}p<0.01$, $^{\#\#\#}p<0.001$), or CH and probiotics-treated group ($^{*}p<0.05$, $^{**}p<0.01$, $^{***}p<0.001$).

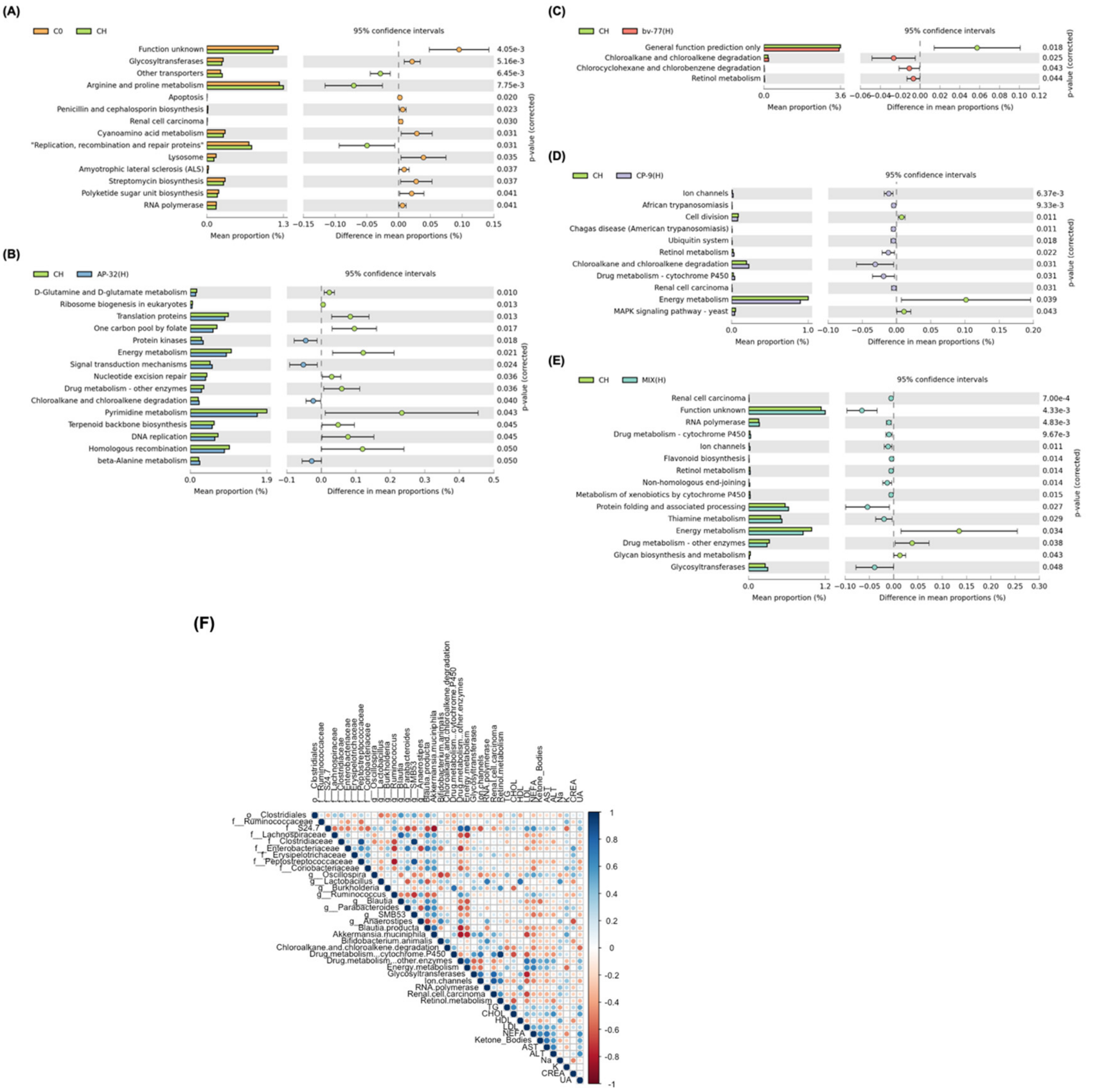

Figure S6. Functional pathways analysis after 8-week probiotic treatment by PICRUSt analysis. Functional pathways analysis was utilized PICRUSt and plotted by STAMP (v2.1.3, https://beiko-lab.cs.dal.ca/software/STAMP, June 26, 2015). (**A~E**) Functional pathways were significantly changed after probiotic treatment. (**F**) Correlation among core microbiota, biochemistry and functional pathways were analyzed by Spearman's correlation.
